# Modelling the effects of precipitation and temperature on malaria incidence in coastal and western Kenya

**DOI:** 10.1186/s12936-025-05428-0

**Published:** 2025-07-01

**Authors:** Amna Tariq, Donal Bisanzio, Francis Mutuku, Bryson Ndenga, Zainab Jembe, Priscilla Maina, Philip Chebii, Charles Ronga, Victoria Okuta, A. Desiree LaBeaud

**Affiliations:** 1 Department of Pediatrics, Division of Infectious Diseases, Stanford University, Stanford, CA USA; 2 Department of Environment and Health Sciences, Technical University of Mombasa, Mombasa, Kenya; 3 Centre for Global Health Research, Kenya, Medical Research Institute, Kisumu, Kenya; 4 RTI International, Washington, DC USA; 5 Vector Borne Disease Control Unit, Msambweni County Referral Hospital, Msambweni, Kenya

**Keywords:** Malaria, Precipitation, Temperature, Generalized additive models, Cross-correlation, Lags

## Abstract

**Background:**

Malaria continues to plague sub-Saharan Africa despite great efforts geared towards its mitigation. In Kenya alone, 70% of the population remains at risk for malaria every year. Malaria is spread by *Anopheles* mosquitoes carrying the *Plasmodium* parasite, and displays a complex ecology with various socio-economic, biophysical factors and meteorological predictors, particularly temperature and precipitation, associated with the occurrence of the disease.

**Methods:**

This study estimated the empirical relationship of temperature and precipitation on the temporal population dynamics of symptomatic malaria cases in Kenyan children living in Ukunda (on Kenyan southern coast), and Kisumu (on Kenyan lake zone) between 2014 and 2022 using daily malaria incidence data collected during a febrile illness surveillance study, merged with daily climatological data collected from ground devices. Generalized additive mixed models (GAMMs) were used to explore the relationship between malaria cases and temperature and precipitation, with Poisson, zero-inflated Poisson and negative binomial distribution and a logarithmic link function. The cross-correlation function assessed the time lags with peak correlations between malaria incidence, precipitation and temperature.

**Results:**

The data showed 673 positive malaria incident cases amongst children in Kisumu compared to 1209 cases in Ukunda. The results indicate a positive correlation of malaria incidence with rainfall and temperature in Kisumu and a positive correlation between malaria incidence and rainfall and a negative correlation between malaria incidence and temperature in Ukunda. The lags between malaria incidence and rainfall were similar for Kisumu and Ukunda and estimated between 7 and 15 weeks. With a time lag of 15 weeks in Ukunda, GAMM depicted a steady relationship between rainfall and malaria cases until rainfall reaches 150 mm and the relationship between malaria cases and temperature peaks at 26–27 °C. In Kisumu using a time lag of 15 weeks in the GAMM, a steady relationship between rainfall and malaria cases was observed until almost 120 mm of rainfall, peaking at 160 mm of rainfall and the relationship between malaria cases and temperature remained steady between 22 and 30 °C.

**Conclusion:**

Assessing the changes in malaria case incidence due to changing seasonality and weather patterns provides policymakers with updated information to strategize malaria control policies.

**Supplementary Information:**

The online version contains supplementary material available at 10.1186/s12936-025-05428-0.

## Background

Despite great efforts geared towards its control, malaria, a climate-sensitive vector-borne disease, continues to affect sub-Saharan Africa causing an estimated 200 million cases and 403,000 deaths, 80% of which are children younger than 5 years [1]. Although substantial progress has been made to eradicate malaria, malaria remains a significant challenge to public health and economic development in Africa [2]. In Kenya alone, the malarial burden accounts for an estimated 13–15% of outpatient consultations [3]. Although climate is not the primary driver affecting the epidemiology and geographic distribution of malaria, it influences malaria through multiple different pathways [4]. Fluctuating temperatures and erratic rainfall influence malaria transmission, particularly in Africa, where the borders of the malarial belt are largely determined by climatic factors that limit the suitability for its vector, the *Anopheles* mosquito, and the *Plasmodium* parasite [5–7]. The mean annual temperature in Kenya has increased by 1.0 °C with an average rate of 0.21 °C per decade since the 1960s and will continue to further increase by 0.8–1.5 °C by the 2030s, enabling the vector to spread to new territory [8].

Malaria displays complex transmission dynamics with various ecological, socio-economic, and biophysical factors impacting the occurrence of the disease. Prior studies have described environmental and climatic factors impacting epidemics of malaria in sub-Saharan Africa which accounts for 95% of malaria cases [9, 10]. Interactions between human hosts, mosquito vectors and plasmodium parasites are conditioned by climate [4]. Studies have highlighted how changes in climatic conditions affect malaria transmission primarily through changes in the life cycles of mosquitoes and parasites, as quantified by specific models [9, 11]. Most of the studies have focused primarily on temperature and rainfall and how these factors affect the vector mosquito population but with somewhat conflicting results about the interpretation of how the variability of climate factors affects malaria incidence in a linear or non-linear pattern [12]. Conflicting opinions exist about the relative importance of meteorological factors affecting malaria transmission in Kenya, attributable to various co-variates such as drug resistance, methodological issues, and climate data collection issues [9, 11, 13]. However, warming temperatures increase the lifespan of *Anopheles* mosquito and the rate of development of the *Plasmodium* parasite. At the same time changes in precipitation and/or humidity create suitable breeding conditions for *Anopheles* mosquitoes leading to an increase in mosquito density and abundance [14]. Climatic factors can serve as important drivers of malaria epidemics [15, 16]. Prior studies have suggested that peak malaria incidence occurs between 25 and 27 °C [17]. A suitable relative humidity of 50–60% has been shown to prolong mosquito survival and the onset of the rainy season increases vector abundance causing increased malaria cases [17]. Other predictors such as deforestation, altitude, farming, socioeconomic status, human mobility, and urbanization have also been identified as important factors that can influence malaria transmission in a region [18–22].

Despite the predicted relationship between ambient temperature, precipitation, and malaria transmission in Africa, understanding the changing weather patterns and their impacts on the geographical shifts of malaria incidence is important to optimize future malaria control interventions and inform malaria elimination programs [23]. As climate continues to change, shifts in geographic locations suitable for malaria transmission are observed, and differing lengths of seasons of suitability are recorded, which will require changes in the types and amounts of resources allocated to malaria control. Malaria transmission is documented to be sensitive to short-term changes in weather conditions, particularly temperature and precipitation [24].

The purpose of the current study is to estimate the short-term empirical relationship of temperature and precipitation on the population dynamics of symptomatic malaria in Kenyan children over time. The association of malaria case incidence with temperature and rainfall in Kenya was analyzed between 2014 and 2022 using daily malaria incidence data collected during a febrile illness surveillance study. This study aims to investigate and compare the relative effects of climate variability on the burden of malaria in coastal and inland Kenya. We examine the seasonal patterns of rainfall and temperature and identify periods when predictable patterns of rainfall and temperature fade, and their correlation with malaria incidence. In this study, time series analysis was used to provide valuable insights into the seasonal patterns of malaria transmission and the influence of weather on malaria patterns in Kenya. The analysis presented here highlights the heterogeneity in seasonal patterns in malaria as detected by passive fever surveillance (2014–2018) and longitudinal cohort surveillance (2019–2022) on the coast (Ukunda) and west (Kisumu) of Kenya, driven by weather, potentially in combination with local ecology, biological and socio-economic factors.

## Methods

### Ethics statement

The study protocol was approved by the Stanford University Institutional Review Board (Protocol ID #31488) and the Kenya Medical Research Institute (KEMRI) National Scientific and Ethical Review Committee (SSC #2611). Written consent was obtained from all participants to collect blood samples. Parents and guardians provided written consent on behalf of children (0–7 years) and children > 7 years of age also provided a written assent. Mature minors provided written consent.

### Study sites

**Fig. 1:**
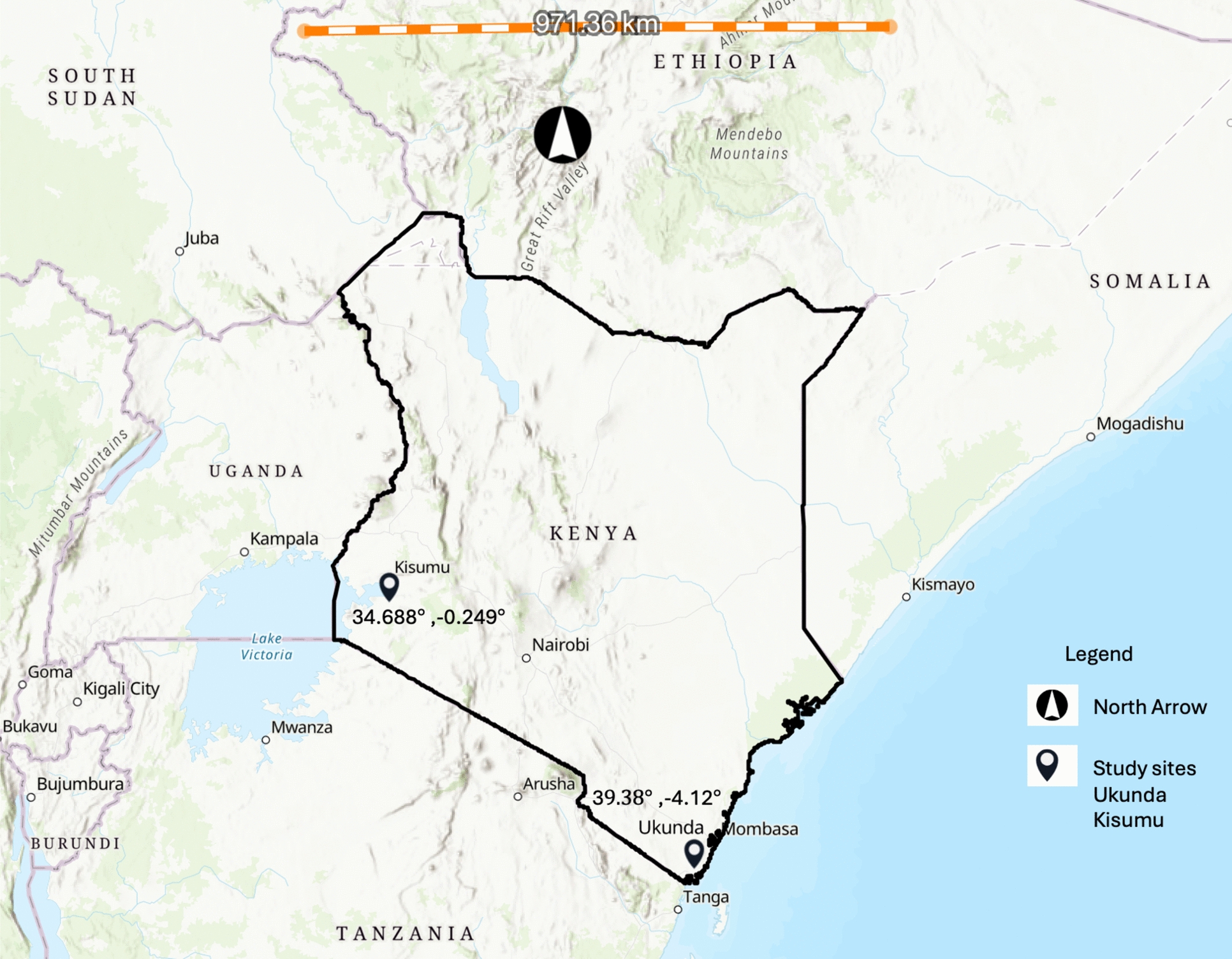
Map of Kenya showing the study sites, created in ArcGIS using the topographic map as the base map; https://www.arcgis.com/home/item.html?id=7378ae8b471940cb9f9d114b67cd09b8

This study examines climate and malaria data from two urban sites in Kenya where human case data and climate data were collected (NIH R01 AI102918: ADL). The study was conducted in two urban study sites that have heterogeneous malaria transmission, Kisumu [− 0.091702°, 34.767957°] on the west and Ukunda [− 4.289796°, 39.567371°] on the coast of Kenya (Fig. 1). The study sites vary in malaria burden, climate, geography, population size, and urbanization [25]. The western study site was at a higher altitude (1131 m above sea level) compared to the coastal site which was near the sea level. The typical Kenya climate is characterized by monsoonal ‘long rains’ (April–June, LRS) and ‘short rains’ (October–December, SRS) rainy seasons, and by hot (January–March, HDS) and cool (July–September, CDS) dry seasons. Although rains are more frequent during the rainy seasons, rains also fall during the dry seasons, increasingly so with climate change-induced weather variation. In general, coastal Kenya is dry and hot but the breeze from the ocean helps to maintain the temperature [26]. The western sites had over two times higher 30-day cumulative rainfall compared to the coastal sites (221 mm in Kisumu and 98 mm in Ukunda). While seasonal temperature variation occurred at both sites, the mean temperature and range was 25.44 °C (13.9–33.5 °C) in Kisumu and 27.75 °C (21.7–41.6 °C) in Ukunda. For the mosquito prevention practices, we found that children visiting the clinic in Kisumu had a lower percentage of window screen (18.8% versus 73.3%) and bednet use (41.9% versus 89.2%) compared to the children in Ukunda. The distribution of assets and other home characteristics varied across sites with a greater number of people belonging to higher socioeconomic strata in Kisumu (54.7%) compared to Ukunda (3.04%). Details of the study cohort can be found in prior studies [27].

### Climate data

The current climate data set is composed of meteorological daily data on mean, maximum, and minimum temperature ($^\circ C)$ and precipitation (mm/day), collected from ground devices in Kenya at the study sites in Kisumu and Ukunda from April 2014 to February 2022. Any missing data on temperature and rainfall was imputed using readings collected and published readily by the “Visual Crossings” which is an easy-to-use, low-cost source of historical and forecast global weather data [28]. The climate data on temperature and precipitation was temporally aggregated to weekly data to avoid over-parametrization, overfitting, and calculation problems faced with analysing daily climate data.

### Malaria case data

A pediatric cohort comprised of children less than 18 years old, who were febrile (axillary temperature of 37.8 °C or higher) at the time of diagnosis and confirmed for malaria (*Plasmodium* spp. infection) using a standard, quality-controlled, Giemsa-stained, blood smear technique performed by trained parasitology technicians, spanning from January 6, 2014- February 22, 2022, was identified, cleaned and analyzed [29, 30]. The immune system and immunological responses of children (defined by the CDC, as a human being below 18 years if age) undergo significant changes and maturation during the age of 0–18 years, making them vulnerable to infection. Moreover, malaria is getting more prevalent in older children (ages 5–15 years) and they are more likely to not report any symptoms, therefore, studying older children between ages 6–18 years is warranted [31]. Malaria presentation in the age group of 5–15 years serves as a source of onward parasite transmission, undermining malaria elimination efforts [31].

In Kenya, improved malaria testing practices have indicated that a high proportion of febrile cases are not linked to *Plasmodium* infection [32]. Many diseases that present in sub-Saharan Africa can manifest malaria-like symptoms and only testing can be done to make an accurate diagnosis and prescribe treatment [30]. For this study, only febrile illness cases positive by microscopy were defined as malaria. Any child who presented with fever (axillary temperature of 37.8 °C or higher) at the time of malaria diagnosis was considered to be a malaria case for this study. Children were enrolled either as member of a longitudinal cohort (January 2014–December 2018 and December 2019–February 2022) or by passive surveillance during times of illness at the local clinic (January 2014–December 2018 only). From March 2020 through June 2020, the case detection and enrollment activities were halted on-site due to the COVID-19 pandemic. As a part of the passive surveillance during times of illness from January 2014 to December 2018, child participants who were less than 18 years old, presenting with acute febrile illness (≤ 5 days) and no localizing symptoms were enrolled at the Ukunda Health Center and Obama Children’s Hospital in Kisumu as a part of the ongoing acute febrile illness surveillance study. All the study participants were evaluated by study physicians, and management decisions, including treatment and disposition, were made at the discretion of the care providers in accordance with local practices in the region [33]. As a part of the 2 longitudinal cohorts (January 2014–December 2018 and December 2019–February 2022) the study team followed children between ages 2–18 years every six-month interval with blood collection, serologic testing (dengue virus and Chikungunya virus IgG), and malaria testing at each follow-up visit. Follow-up visits were conducted door-to-door on occasion and at a central facility to interact with the study participants. Study participation was voluntary. There were dropouts during the study period over time and all visits were scheduled regardless of symptoms, with the majority qualifying as healthy visits [34]. Daily malaria case data was aggregated into weekly malaria case data in coherence with the weekly temperature and precipitation data.

### Data analysis

Several descriptive summaries (mean, minimum, and maximum) were considered for the two main covariates: temperature and precipitation. Distribution of the mean, minimum, and maximum temperature and precipitation was defined on a daily and weekly temporal scale and patterns of temperature and humidity were identified over time. Then Generalized Additive Mixed Models (GAMMS) were fitted to the outcome predictor, i.e. the malaria case incidence. GAMMS are non-parametric regression techniques, an extension of conventional Generalized Linear Models (GLMs) that allow one to model non-parametric relationships between the response variable and continuous covariates [35]. The GAMM tries to fit the observed data as closely as possible by enabling the smooth effects of the continuous predictors as well as the spatial structure of the data [36]. First, GAMMs were utilized to observe the seasonal patterns in precipitation and mean temperature over time. To model the effect of precipitation and mean temperature on malaria case incidence during 2014–2022 for the two regions, GAMM was fitted using a Poisson distribution and smoothing parameters, that control the trade-off between the fit and smoothness of the model, applied to the weekly malaria incidence and weekly mean temperature and precipitation data. The malaria case incidence is a count variable and could be modeled using count regression models; however, modeling count data presents numerous issues, particularly when the data contains a large number of zeros and is over-dispersed as was the case in the dataset pertaining to this study [37]. Further, the assumptions of independence and equi-dispersion might be inappropriate due to dependence on individual response, heterogeneity, clustering, and exclusion of some causal factors of the disease [38, 39]. The problem of over-dispersion in modeling count data can be solved by fitting negatively binomial, quasi-Poisson, or generalized Poisson regression models. The relationship of malaria case incidence with climate is debatable, and the effects of the climatic covariates are not homogeneous across their ranges and are nonlinear [40]. Data was fitted to GAMMs with count regression models such as Poisson, zero-inflated Poisson distribution, and negative binomial distribution and a logarithmic link function to explore the relationship between malaria cases and climate predictors, i.e., mean temperature and precipitation. The best model was selected using the Akaike Information Criterion (AIC).

A non-parametric seasonal Mann–Kendall test was also performed to detect monthly trends of malaria, mean temperature and precipitation. This test is particularly useful to detect trends in data when underlying distribution is unknown. Mann–Kendall’s rank correlation tau and the associated p-value for temperature and precipitation was investigated [41].

The cross-correlation function between the time series of the weekly precipitation, weekly mean temperature, and the weekly malaria incidence assessed the time lags with peak correlations between malaria incidence and precipitation and mean temperature. These time lags were used in the GAMM of precipitation and mean temperature on the weekly malaria incidence. All analyses were performed in R language (Version 2022.12.0 + 35) R: The R Project for Statistical Computing. Accessed June 26, 2023. https://www.r-project.org/ [42].

## Results

### Malaria cases

**Fig. 2:**
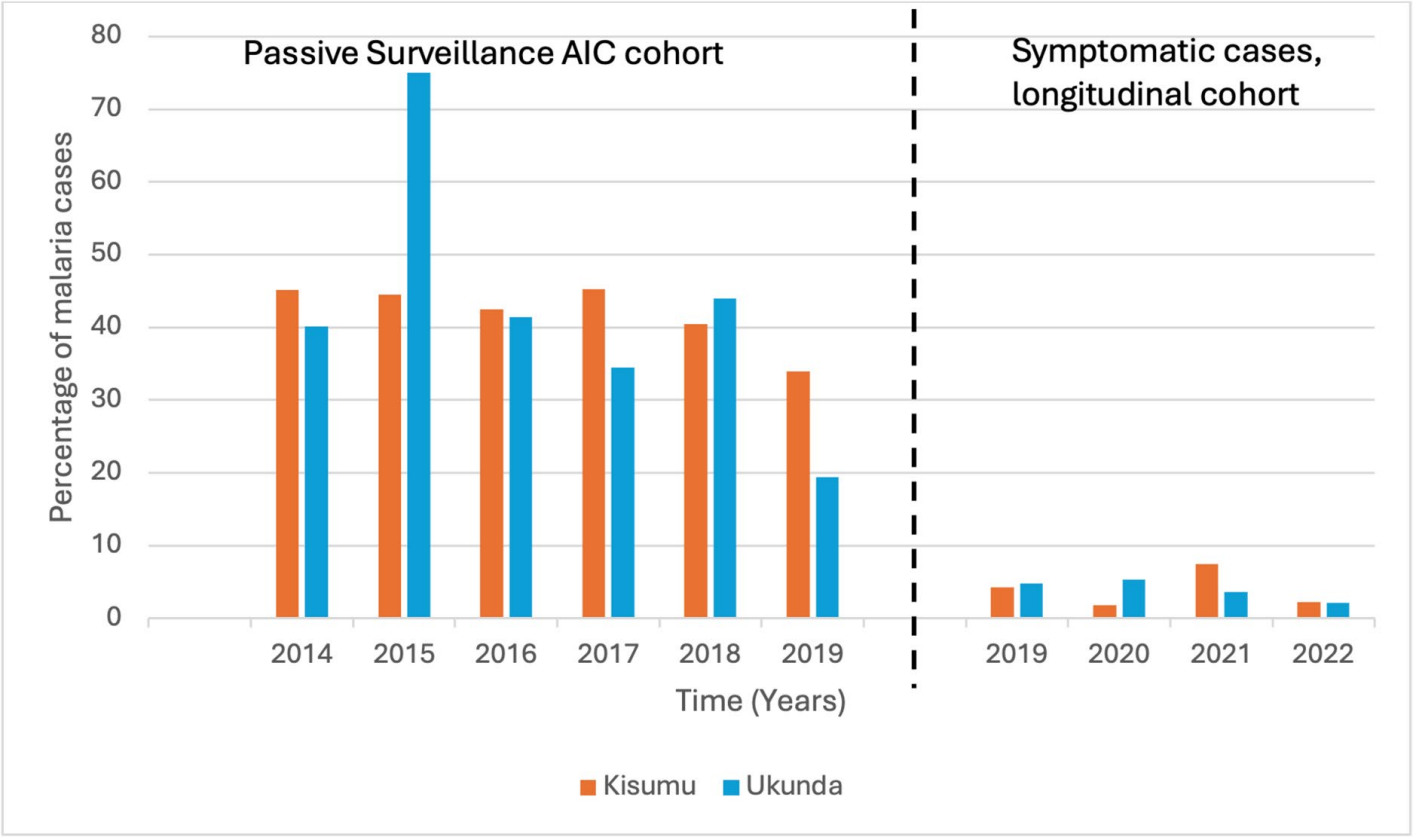
Prevalence (percentage) of symptomatic malaria-positive cases in Kisumu and Ukunda among all febrile study child participants (age 2–18 years) between April 2014 and February 2022 by site. The black dotted indicates the time when the data collection changed from passive surveillance of the AIC cohort to the longitudinal cohort

During the study period, 673 positive malaria incident cases were detected amongst children in Kisumu compared to 1209 cases in Ukunda. During the 2014–2018 period, 603 (7.9%) febrile malaria- positive cases were detected in Kisumu and 1027 (15.9%) cases in Ukunda during the passive surveillance at local clinics. From December 2019-January 2022, 110 (8.7%) febrile malaria- positive pediatric cases were identified in Kisumu amongst the 1271 participants enrolled in the Kisumu cohort and 39 (4.4%) febrile malaria-positive pediatric cases were identified in Ukunda amongst the 886 participants enrolled in the Ukunda cohort. The percentage of malaria-positive symptomatic cases for Kisumu and Ukunda is given in Fig. 2.

### Kisumu, Western Kenya

**Fig. 3:**
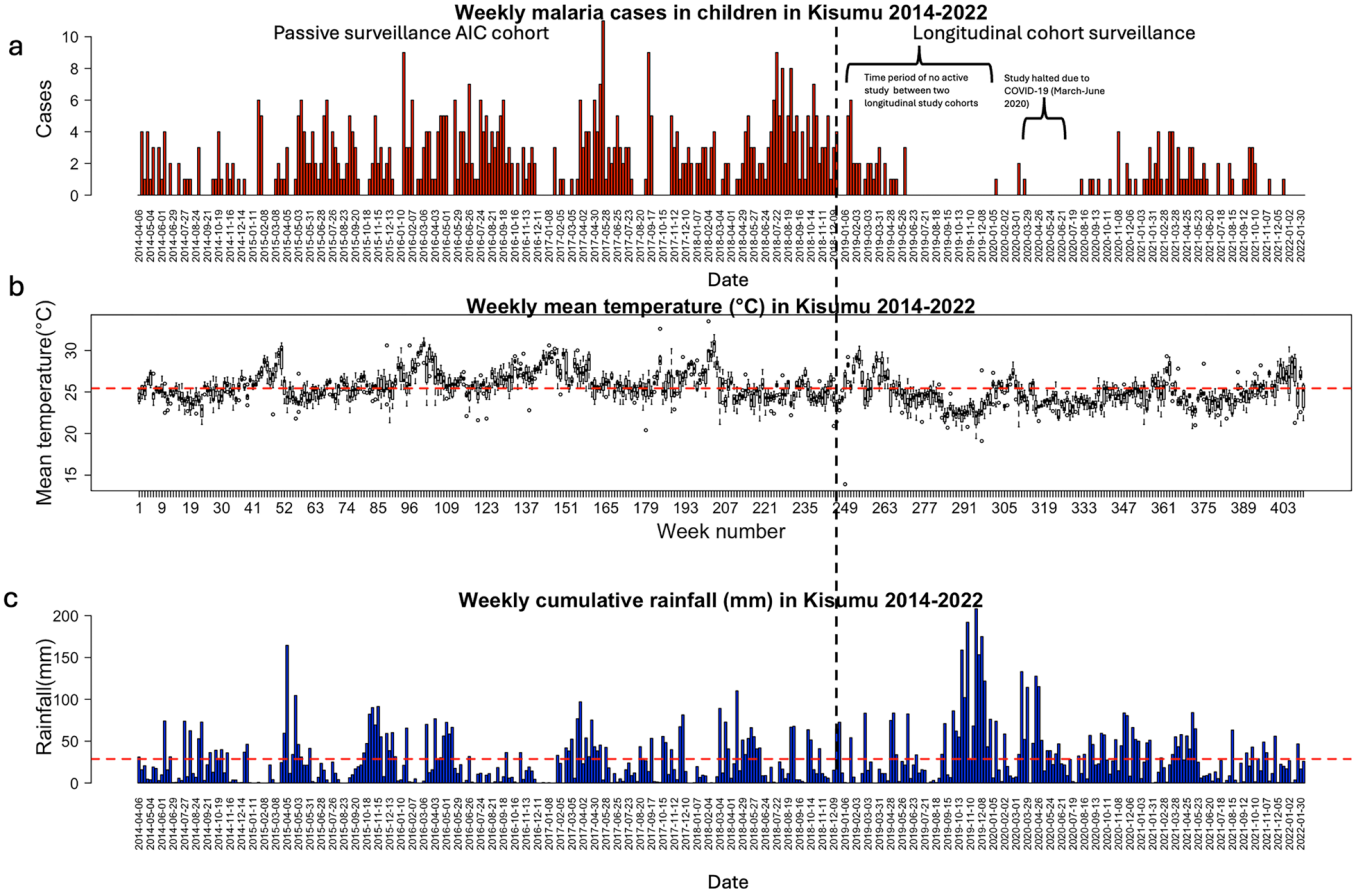
Time series of cumulative rainfall, average mean temperature, and total malaria cases by week in Kisumu from 2014 to 2022. The black dotted line indicates the time when the malaria case collection changed from passive surveillance conducted in the AIC cohort to longitudinal cohort surveillance. **a** Weekly symptomatic malaria cases in Kisumu, 2014–2022. **b** Average mean temperature in Kisumu, 2014–2022 per week. The red dashed horizontal line shows the average weekly mean temperature over the entire study period. **c** Sum of weekly rainfall in Kisumu from 2014 to 2022. The red dashed horizontal line shows the average weekly rainfall over the entire study period

The average weekly mean temperature in Kisumu was calculated to be 25.44 °C (SD: 1.64 °C) and the average weekly rainfall was calculated to be 28.7 mm (SD: 32.5 mm). The average monthly cumulative rainfall in Kisumu (122 mm (SD: 98.5 mm) was higher than the average cumulative monthly rainfall in Ukunda (93.55 mm (SD: 114.75 mm). The time series for average weekly mean temperature showed that between 2014 and 2022, temperature exhibited cyclical patterns, with prominent peaks between the months of February-April each year. Kisumu also has two rainy seasons; the heaviest rains from mid-March to mid-May and the shorter rains from November–December although the rains in December 2019 were much higher. January and February are mostly dry with little chance of rainfall. Figure 3 shows that rainfall remained under 100 mm per week for most of the 8 years, but between April–May 2015 and August 2019-April 2020, excessive rainfall was observed. An increase in weekly malaria cases can be observed 7–15 weeks after an increase in rainfall, such as an increase in cases in February 2016 and July 2018 (Fig. 3). The seasonal Mann–Kendall trend test showed a significant decreasing trend in malaria cases over the study period (z = − 6.04, p < 0.001) as well as a decreasing trend in mean temperature (z = − 12.06, p < 0.001). An increasing trend in precipitation was observed over the study period (z = 3.5, p < 0.001).

The weekly malaria incidence lagged 7–15 weeks behind the weekly precipitation (Supplementary File Fig. 1). The cross-correlation function showed a positive correlation between rainfall and malaria incidence in Kisumu, indicating that a higher amount and intensity of rainfall results in an increased number of malaria cases. As low case numbers for malaria were observed between 2019 and 2022 due to cohort surveillance only, the cross-correlation function was calculated until the year 2019. We find a relevant influence of proceeding rainfall events on malaria incidence. As observed with rainfall, a positive correlation was observed between mean temperature and malaria cases in Kisumu. Mean temperature was positively correlated to the malaria incidence at lags of 3–4 months. (Supplementary File Fig. 2).

Applying the GAMM to the monthly rainfall data in Kisumu, a cyclical pattern of rainfall was observed, fluctuating between 1.5 and 6 mm from April 2014 to October 2018, peaking in August–September each year, after which the rainfall increases to 8 mm by June 2020 and then declines again until February 2022 (Supplementary File Fig. 3). However, when GAMM is fitted to the cumulative rainfall data at a finer scale, between April 2014 and July 2019, a cyclical pattern in rainfall is observed, with the peak occurring between March and July each year. A disruption in the cyclical pattern was seen in 2015, with maximum rainfall occuring in December 2015 and thereafter another peak in the rainfall is not observed until March 2017. The GAMM predicts an increase in rainfall of more than 10 mm after July 2019, whereas from April 2014 to July 2019, the rainfall remains between 0 and 7 mm (Supplementary File Fig. 4). The temperature shows a sinusoidal pattern from April 2014 to February 2022 with the highest reported temperature of 27 °C in October 2016 and the lowest reported temperature of 24.2 °C in April 2020 (Supplementary File Fig. 5).

**Fig. 4:**
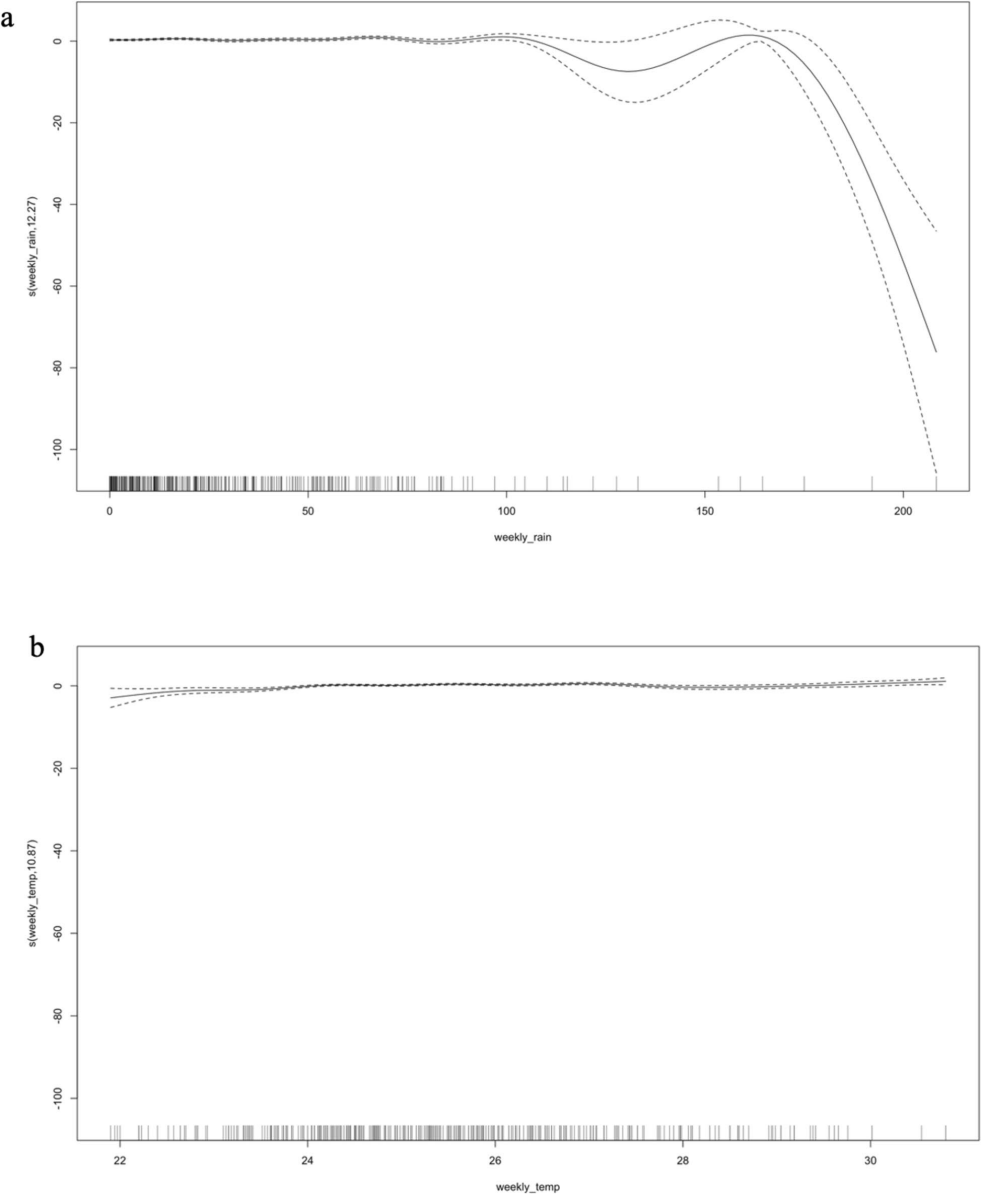
GAMM fitted to the rainfall data with a smoothing factor of 14 (**a**) and mean temperature data with a smoothing factor of 14 (**b**) and malaria case incidence as the output in Kisumu. The black solid line shows the mean fit of the model and black dashed lines show the 95% CI for the mean model fit

From the cross-correlation analysis conducted for precipitation and malaria case incidence and mean temperature and malaria case incidence in Kisumu, time lags of 2–4 months can be applied to the GAMM. When we use a time lag of 15 weeks in the GAMM with both rainfall and mean temperature as the predictors, we observe that there is a steady relationship between rainfall and malaria cases until almost 120 mm of rainfall, peaking at 160 mm of rainfall and declining thereafter (Fig. 4A). The relationship between malaria cases and mean temperature remains steady between 22 and 30 °C (Fig. 4B). The coefficients of the GAMM can explain 13.9% deviance in the model and 5.9% incidence variation (R^2^ = 0.059) with both smoothing terms being statistically significant (p < 0.05). The effective degrees of freedom (edf) were greater than two for both rainfall and temperature showing a higher non-linear relationship of malaria cases with the climatic predictors, suggesting that the spline is distinguishable from the straight line.

### Ukunda, coastal Kenya

**Fig. 5:**
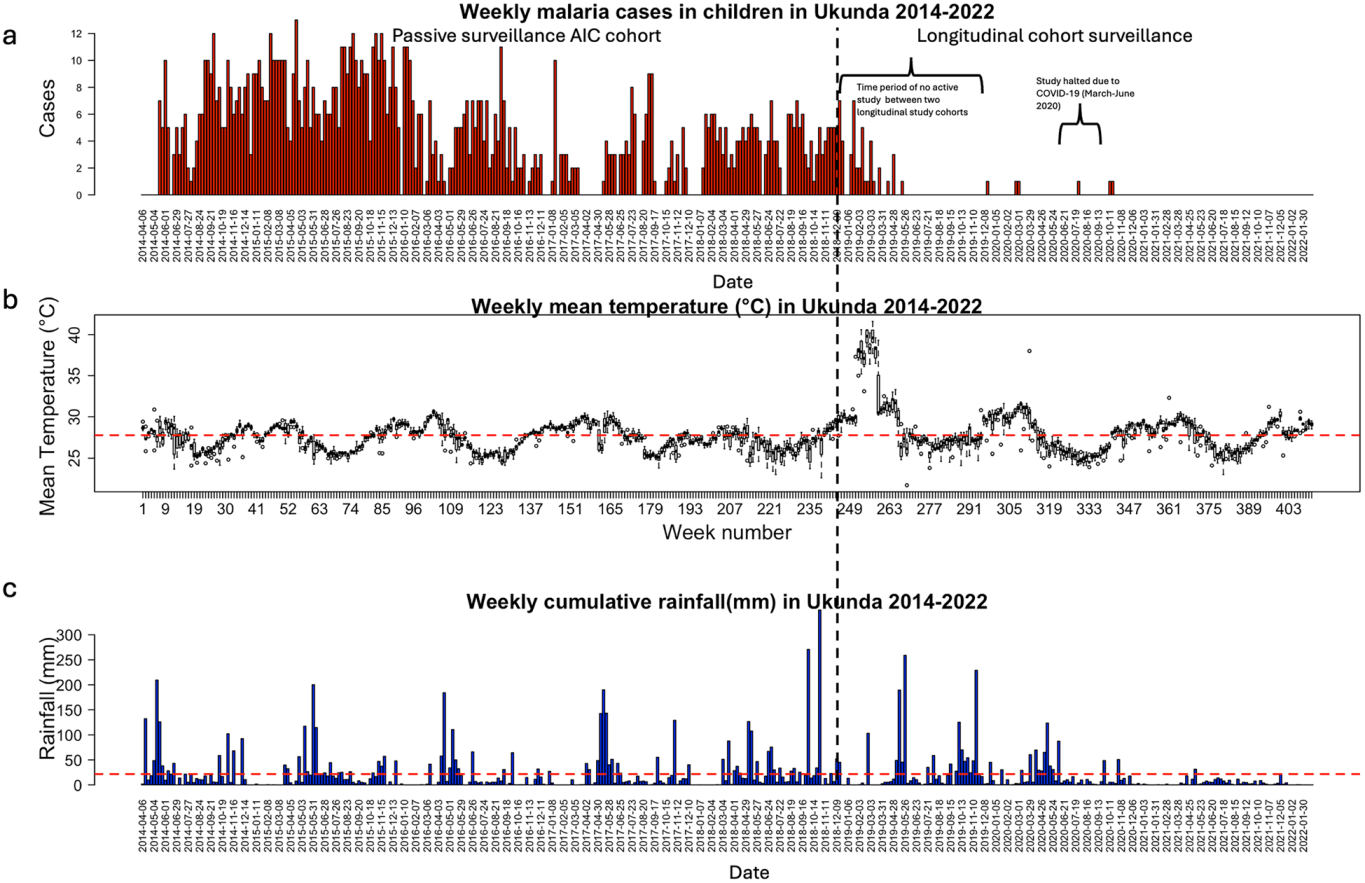
Panel plot showing the weekly rainfall, mean temperature, and malaria case incidence in Ukunda, Kenya. The black dotted line indicates the time when the malaria case collection changed from passive surveillance conducted in the AIC cohort to longitudinal cohort surveillance. **a** Weekly symptomatic malaria cases in Ukunda, 2014–2022. **b** Average mean temperature in Ukunda, 2014–2022 per week. The red dashed horizontal line shows the average weekly mean temperature over the entire study period. **c** Sum of weekly rainfall in Ukunda from 2014 to 2022. The red dashed horizontal line shows the average weekly rainfall over the entire study period

The average weekly mean temperature in Ukunda was 27.75 °C (SD: 2.20 °C), ~ 2 °C higher than the average weekly temperature in Kisumu and the average weekly rainfall was 21.57 mm (SD: 41.77 mm), lower than Kisumu. Ukunda has two rainy seasons: the heaviest rains from mid-March to mid-May and the shorter rains from November to December. January and February are mostly dry with little chance of rainfall. The average monthly cumulative rainfall in Ukunda was 93.55 mm (SD: 114.75 mm), 28.5 mm less than in Kisumu. Between 2014 and 2022, the mean temperature showed cyclical patterns, with bimodal peaks in each sinusoidal wave between the months of November and April with extremely high temperatures (35–40 °C) reported from January to March 2019 (Fig. 5). Higher than average rainfall (up to 250 mm) was recorded between May and June of each year over eight years that this study was conducted. An anomaly in the rainfall pattern and quantity was recorded from September to October 2018, measuring up to 350 mm between these months. Generally, higher pediatric malaria case incidence was recorded in Ukunda compared to Kisumu during the passive malaria surveillance. However, fewer cases were reported after May 2019, when the study switched to longitudinal cohort surveillance. An increase in weekly malaria cases can be observed 7–15 weeks after an increase in rainfall (Fig. 5). The seasonal Mann–Kendall test showed an increasing trend in mean temperature (z = 5.4, p < 0.001) and a declining trend in precipitation (z = − 1.26, p = 0.205) and malaria cases (z = − 20.7, p < 0.001) over the study period.

The weekly malaria case incidence lagged 15 weeks behind the weekly precipitation in Ukunda (Supplementary File Fig. 6). The cross-correlation function showed a positive correlation between rainfall and malaria incidence in Ukunda, indicating that a higher amount of rainfall would result in an increased number of malaria cases. As low case numbers for malaria were observed between 2019 and 2022 due to changes in case detection methods, and switching to longitudinal cohort surveillance, the cross-correlation function was calculated until the year 2019. We find a relevant influence of preceding rainfall events on malaria incidence in Ukunda as was observed in Kisumu (Supplementary File Fig. 6). A negative correlation was observed between mean temperature and malaria cases in Ukunda. The mean temperature was negatively correlated to the malaria incidence at lags of 0–4 months (Supplementary File Fig. 7) indicating that higher temperatures decrease the malaria case incidence in Ukunda.

Applying the GAMM to the monthly rainfall data in Ukunda, a sinusoidal wave is observed, fluctuating between 1 and 6.5 mm of rainfall from 2014 to 2022. The rainfall pattern declines from 5 to 3 mm between April 2014 and April 2016, after which we see a slight incline in monthly rainfall until February 2019 after which another steep decline in rainfall pattern can be observed until February 2022 when rainfall almost diminishes to 0 mm (Supplementary File Fig. 7). However, when GAMM is fitted to the rainfall data between the time interval of April 2014–July 2019, it can be seen that the rainfall occurs in an almost steady pattern over five years averaging at 4 mm per month (Supplementary File Fig. 8). If the pattern of rainfall is assessed with the monthly cases and GAMM is fitted to the rainfall data every month until 2019, an increase in case count 2–4 months after the increase in rainfall can be appreciated (Supplementary File Fig. 9). The temperature shows a sinusoidal pattern with the second wave peaking at 28 °C at the end of 2016 and the third biggest wave peaking at 30 °C in May 2019 (Supplementary File Fig. 10).

**Fig. 6:**
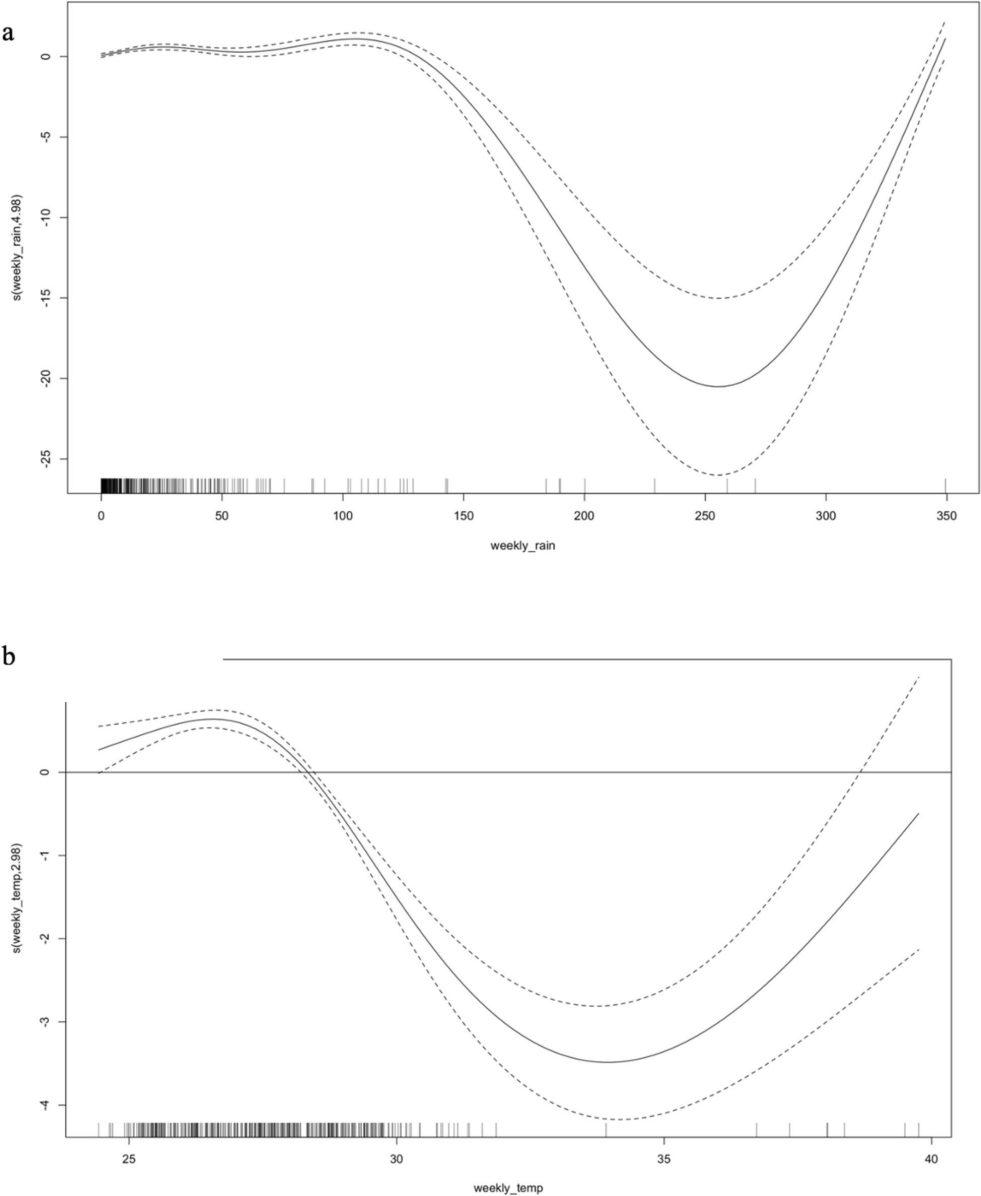
GAMM fitted to the rainfall data with a smoothing factor of 6 (**a**) and temperature data with a smoothing factor of 4 (**b**) and malaria case incidence as the output in Ukunda. The black solid line shows the mean fit of the model and black dashed lines show the 95% CI for the mean model fit

The time lag of 2–4 months obtained from the cross-correlation analysis between precipitation, temperature, and malaria incidence in Ukunda, can be applied to the GAMM. When a time lag of 15 weeks in the GAMM is used with both rainfall and temperature as the predictors of interest, a steady relationship between rainfall and malaria cases can be observed until rainfall reaches 150 mm after which a decline in the relationship occurs (Fig. 6). The relationship between malaria cases and temperature peaks at 26–27 °C. The coefficients of the GAMM can explain 0.9% incidence variation (R^2^ = 0.0094) with both smoothing terms being statistically significant (p < 0.05) (Fig. 6).

## Discussion

Temperature and rainfall have long been recognized as important environmental determinants of malaria [8, 43]. Malaria incidence is impacted by the *Anopheles* vector population, which in turn is impacted by changes in temperature and rainfall. The data showcased the known seasonality, with dry season occurring from January to February repeatedly over the eight years and the rainy season occurring from March to May and November to December repeatedly over the years. The cross-correlation analysis showed an appropriate congruence of malaria incidence and precipitation in Kenya, as is seen in other studies [15, 44]. A 9-week lag was observed in malaria incidence and precipitation in Ghana and rainfall at a time lag of 8 weeks increased malaria transmission in Kenya [15, 44]. In this study, malaria case incidence had the highest positive correlation with total precipitation 7–15 weeks prior. These results are also similar to a study conducted in Zanzibar where the cross-correlation analyses showed that the number of malaria index cases had the highest correlations with total precipitation in the 12th and 13th weeks prior to malaria case confirmation [24]. Another study conducted in Ghana among children aged < 15 years showed a 9-week lag between malaria incidence and precipitation [15]. Studies conducted in Ethiopia and Eritrea reported a rainfall lag of 2 months and a lag of 1–4 months, respectively [45, 46]. The period of ~ 10 weeks coincides with the theoretical vector-parasite-host cycle of the three organisms involved under optimum conditions, assuming that the first blood meal of *Anopheles* is on an infected human and that the temperature is at mean ≥ 25 °C [15]. Other investigators have shown a strong temporal link between rainfall and malaria case incidence. In Eastern Sudan, rainfall was a significant climatic variable in the transmission of malaria [47]. In China, increasing monthly malaria incidences were positively correlated with monthly mean climatic variables including relative humidity, temperature, and precipitation, with a lag effect of one month [48].

This study highlighted a positive cross-correlation of malaria and temperature in Kisumu, which sits at the optimal temperature for malaria transmission, whereas a negative correlation between malaria and temperature was observed in Ukunda, which is hotter than the temperature optimum for malaria, at multiple different lags between 0 and 3 months. The malaria case incidence increased with a rise in temperature in Kisumu, whereas the malaria case incidence decreased with an increase in temperature in Ukunda. This could be due to the temperature that has a critical role in the regulation of growth, development, and survivor-ship of mosquito and malaria parasites, and also determines the period of the gonotrophic cycle [46]. A negative association between temperature and malaria burden has also been reported from western Ethiopia at several lags from 0 to 4 months [12, 49]. As explored in prior studies, hotter and warmer temperatures do not always imply an increase in the burden of malaria in the region [23]. In fact, Shah et al. described the thermal optima for malaria to peak at 25 °C amongst children [25]. As temperatures rise, the relative probability of infection shifts towards arboviruses that have a higher thermal optimum [23].

Model fitting to Kisumu and Ukunda climate and case data indicated that environmental factors including precipitation and temperature had a significant smoothed (non-linear) relationship since their *p*-values were less than 0.05. The effective degree of freedom (edf), the summary statistic of GAMM, showed that monthly malaria cases had a higher non-linear relationship with climate variables since their edf was greater than two. This suggests that climate warming and malaria incidence do not follow a linear pattern such that when temperature rises, malaria case incidence also rises. Similar non-linear relationships between temperature and malaria case incidence have been cited by prior studies [25, 50]. The findings from fitting the GAMM in a study from northwest Ethiopia also suggest climate and environmental parameters including precipitation and temperature to have a significant nonlinear impact on spatiotemporal malaria incidence. However, there is a need to understand the contribution of spatial parameters on malaria transmission. Meanwhile, the malaria transmission encompassing ranges of rainfall, minimum temperature, and maximum temperature was consistent with research findings suggesting that malaria transmission is associated with climate variability [51, 52]. In Ukunda, the relationship between malaria cases and temperature peaked at 26–27 °C, confirming prior data [25, 43, 53]. The Mann–Kendall test indicated an increasing trend in mean temperature in Ukunda consistent with prior studies revealing warming of the region [54]. However an increasing trend in precipitation was seen in Kisumu and a decreasing trend in precipitation was seen in Ukunda. These mixed findings are consistent with the existing literature [54]. A study by Abuya et al. reported declining rainfall in a west sub-county of Kenya [55], whereas certain counties in Kenya receive more rainfall, hence upward trend in precipitation as seen in Kisumu [54].

### Limitations

While this study adequately explains the spatial–temporal association of precipitation and temperature with malaria case incidence on the coast and west of Kenya, this study has some limitations. The malaria case incidence data used in this study was not acquired from the national surveillance system. The incidence data was extracted from the longitudinal cohort study. During 2018–2022, the method of surveillance switched, and even lower malaria case numbers were reported in our data. On both the coast and west of Kenya, the majority of malaria cases were reported during 2014–2018 during clinic-based passive fever surveillance of symptomatic cases. Only malaria smear test results were utilized to include participants in the study and, therefore, no conclusions can be drawn on the impacts of climate on sub-parasitemic malaria. It is not possible to completely quantify how climate change alone affects malaria transmission, which interacts with many factors such as population and demographic dynamics, drug resistance, insecticide resistance, human activities, such as deforestation, irrigation, swamp drainage, and their impact on the local ecology.

## Conclusions

In conclusion, rainfall and temperature have a positive correlation with malaria incidence in Kisumu and a positive correlation between malaria incidence and rainfall, and a negative correlation between malaria incidence and temperature in Ukunda. The relationship between malaria incidence and rainfall was complex and it was not directly linear. The lags between malaria incidence and rainfall were similar for Kisumu and Ukunda and estimated between 7 and 15 weeks. This study shows that a deeper understanding of climate change and malaria transmission patterns is required to inform and optimize malaria control policies and public health decisions by health policymakers. An expansive understanding of malaria seasonality in Kenya can inform malaria control and elimination measures, leading to targeted timing of bed-net distribution or indoor residual spraying to ensure maximum efficacy. Assessing the changes in malaria case incidence due to changing seasonality and weather patterns provides policymakers with updated information to strategize malaria control policies.

## Supplementary Information

Additional file 1

## Data Availability

The datasets used and/or analysed during the current study are available from the corresponding author on reasonable request.

## Abbreviations

GAMMs: Generalized additive mixed models
LRS: Long rainy season
SRS: Short rainy season
HDS: Hot dry season
CDS: Cool dry season
GLMs: Generalized linear models
AIC: Akaike information criteria
Edf: Effective degrees of freedom
