# Supplementary material for: Modelling the effects of precipitation and temperature on malaria incidence in coastal and western Kenya
Source: Malar J. 2025 Jul 1;24:208. doi: 10.1186/s12936-025-05428-0 (PMC12217918; doi:10.1186/s12936-025-05428-0)
Supplement: Supplementary file 1 — Additional file 1 [file 12936_2025_5428_MOESM1_ESM.docx]

**Modeling the effects of precipitation and temperature on malaria incidence in coastal and western Kenya**

Amna Tariq MPH, PhD^1*^, Francis Mutuku PhD^2^, Bryson Ndenga PhD^3^, Donal Bisanzio DVM PhD^4^, Zainab Jembe^5^, Priscilla Maina^5^, Philip Chebii^5^, Charles Ronga^3^, Victoria Okuta^3^, A. Desiree LaBeaud MD MS^1^

^1^Department of Pediatrics, Division of Infectious Diseases, Stanford University, Stanford, CA, United States,

^2^Department of Environment and Health Sciences, Technical University of Mombasa, Mombasa, Kenya,

^3^Centre for Global Health Research, Kenya, Medical Research Institute, Kisumu, Kenya,

^4^RTI International, Washington, DC, USA,

^5^Vector borne Disease control Unit, Msambweni County Referral hospital, Msambweni, Kenya.

Corresponding author: Amna Tariq (atariq1@stanford.edu)


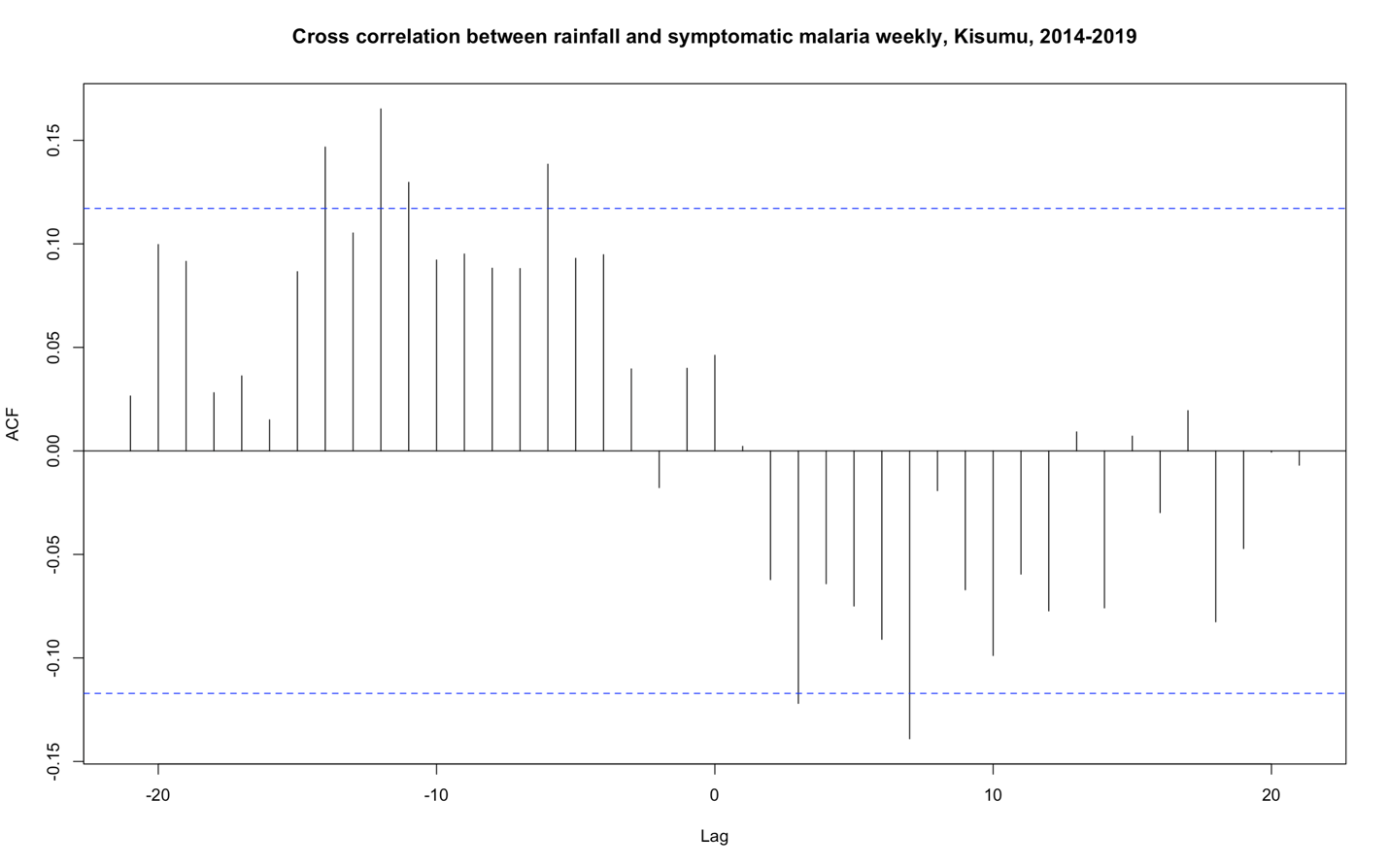


Fig 1. Cross correlation function between weekly cumulative rainfall and symptomatic malaria cases 2014-2018 in Kisumu. Malaria cases occur after 7-15 weeks of rainfall, showing a positive relation between rainfall and malaria cases.


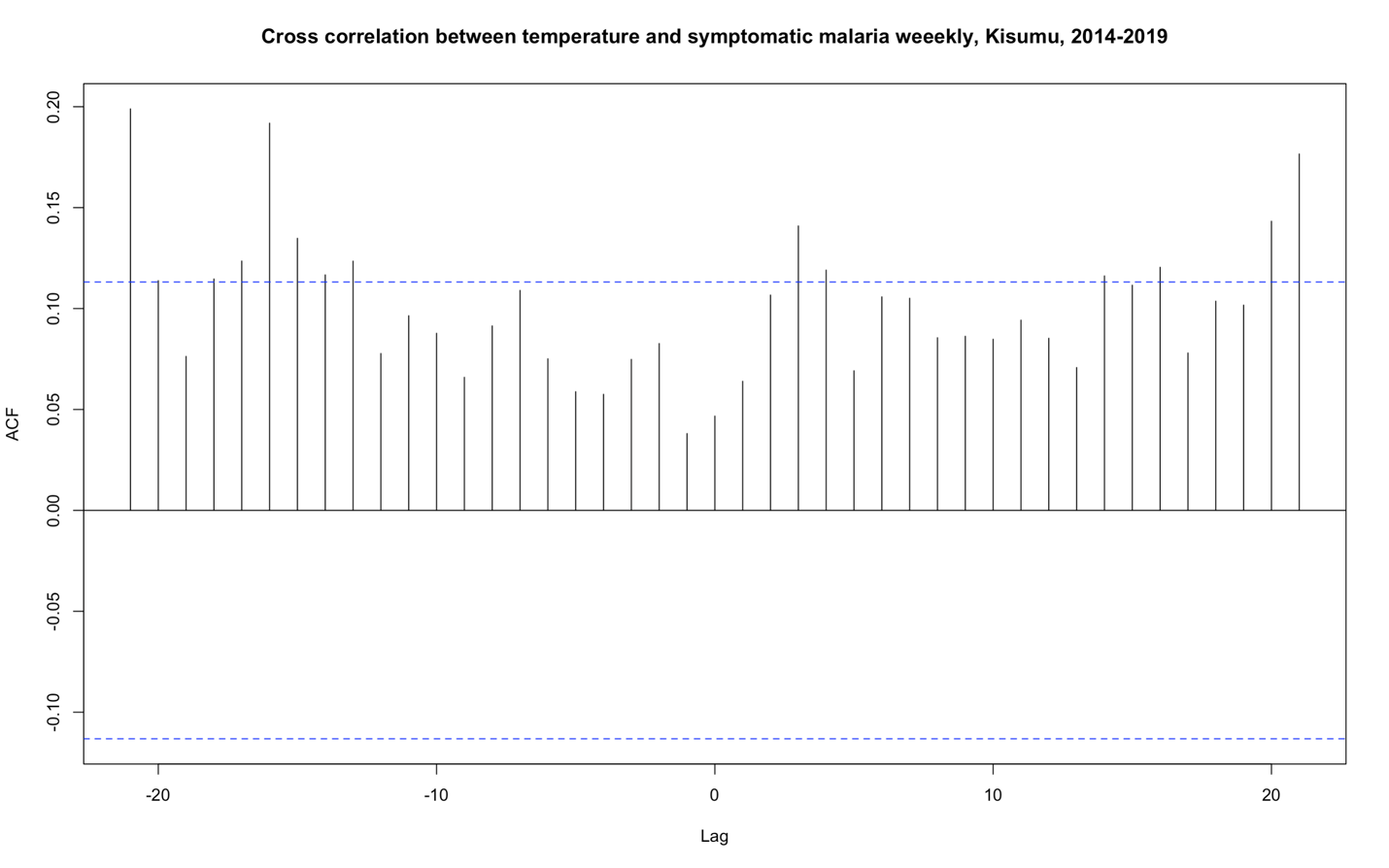


Fig 2: Cross correlation function between weekly average temperature and malaria cases

2014-2018 in Kisumu. Malaria cases occur after a lag of 15-20 weeks in temperature changes. A positive correlation is observed between temperature and malaria cases, indicating increasing temperature related to increase in malaria cases.


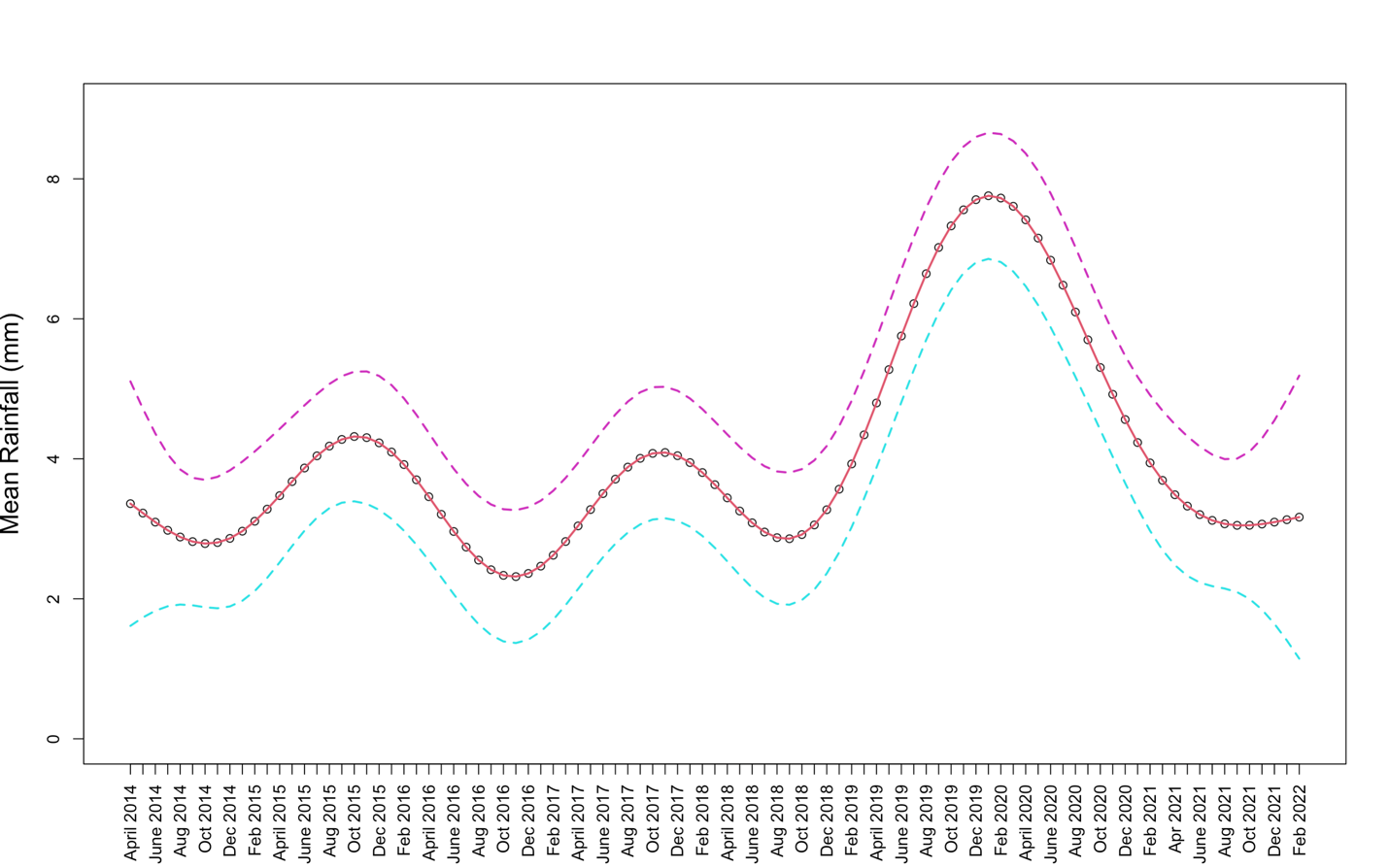


Fig 3: Generalized Additive Model fit to the rainfall data by the month in Kisumu 2014-2022. The red line shows the mean fit of the model and the magenta and blue dashed lines show the 95% CI for the mean model fit.


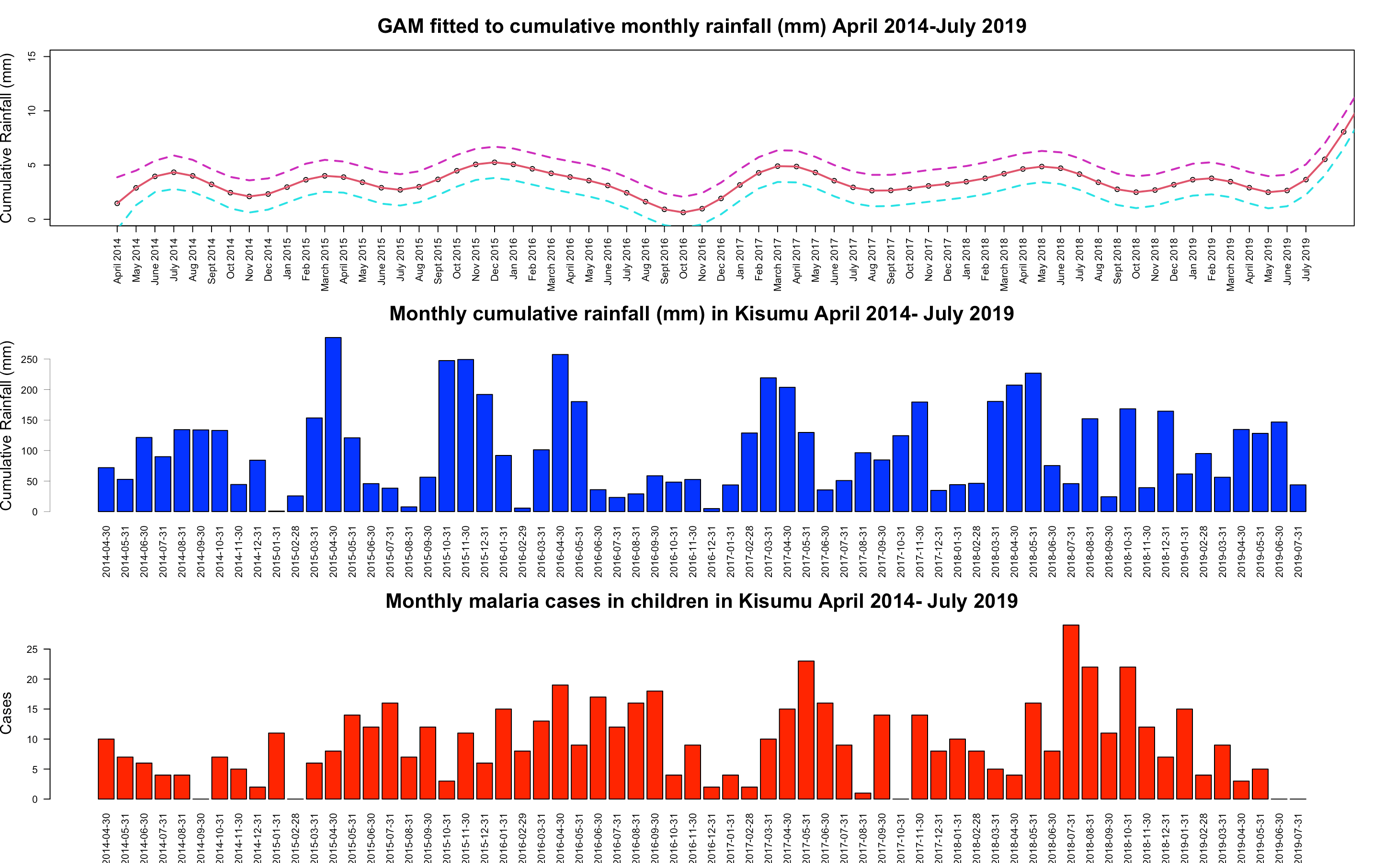


Fig 4 : Panel a: Generalized Additive model (GAM) fitted to the cumulative rainfall data April 2014-July 2019.

Panel b: Monthly cumulative rainfall April 2014-July 2019.

Panel c: Monthly malaria cases April 2014-July 2019.


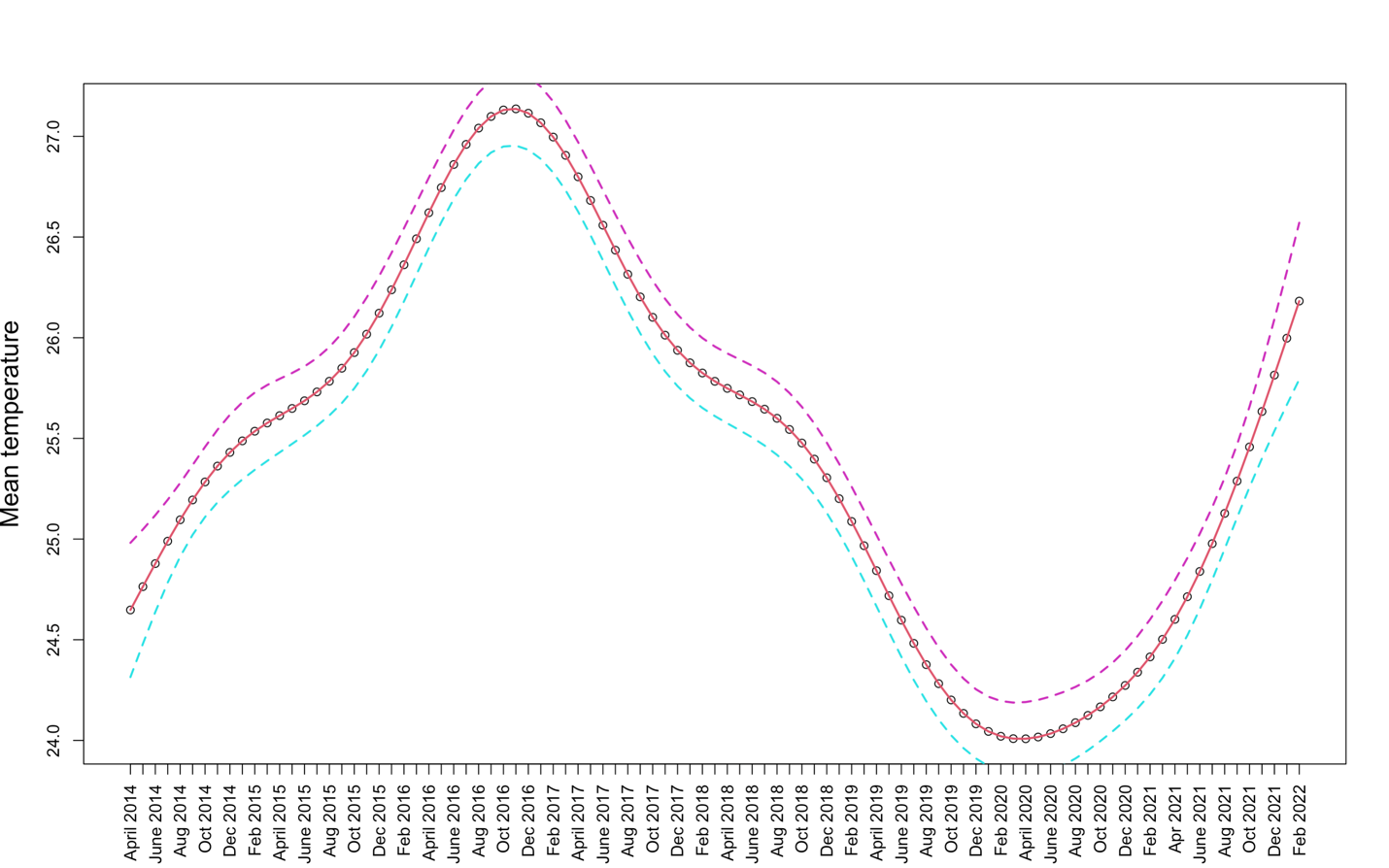


Fig 5: Generalized Additive Model fitted to the mean temperature data by months. The red line shows the mean fit of the model and the magenta and blue dashed lines show the 95% CI for the mean model fit.


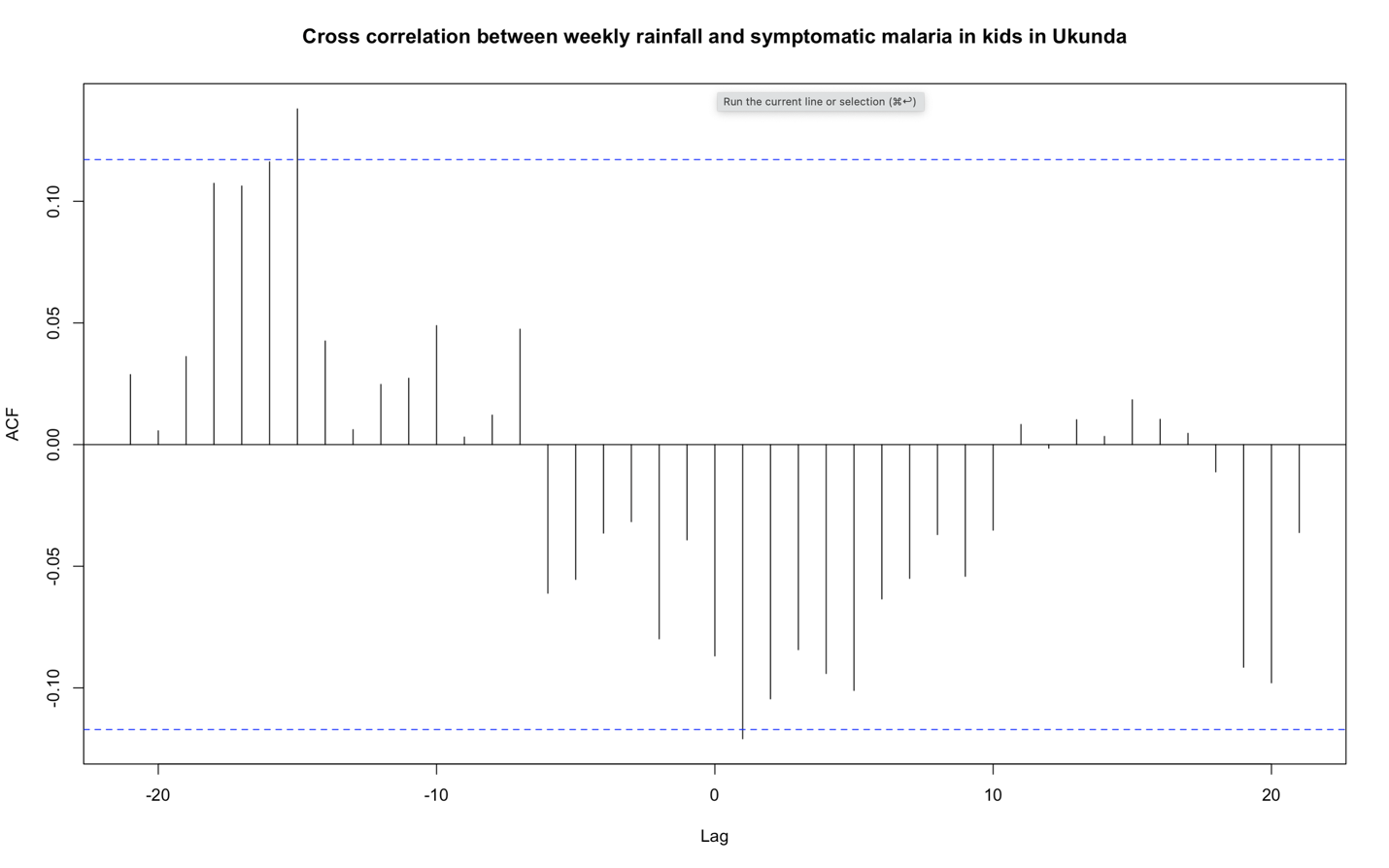


Fig 6: Cross correlation function between weekly rainfall and symptomatic malaria cases April 2014- July 2019 in Ukunda. Malaria cases occur after 15 weeks of rainfall, showing a positive relation between rainfall and malaria cases.


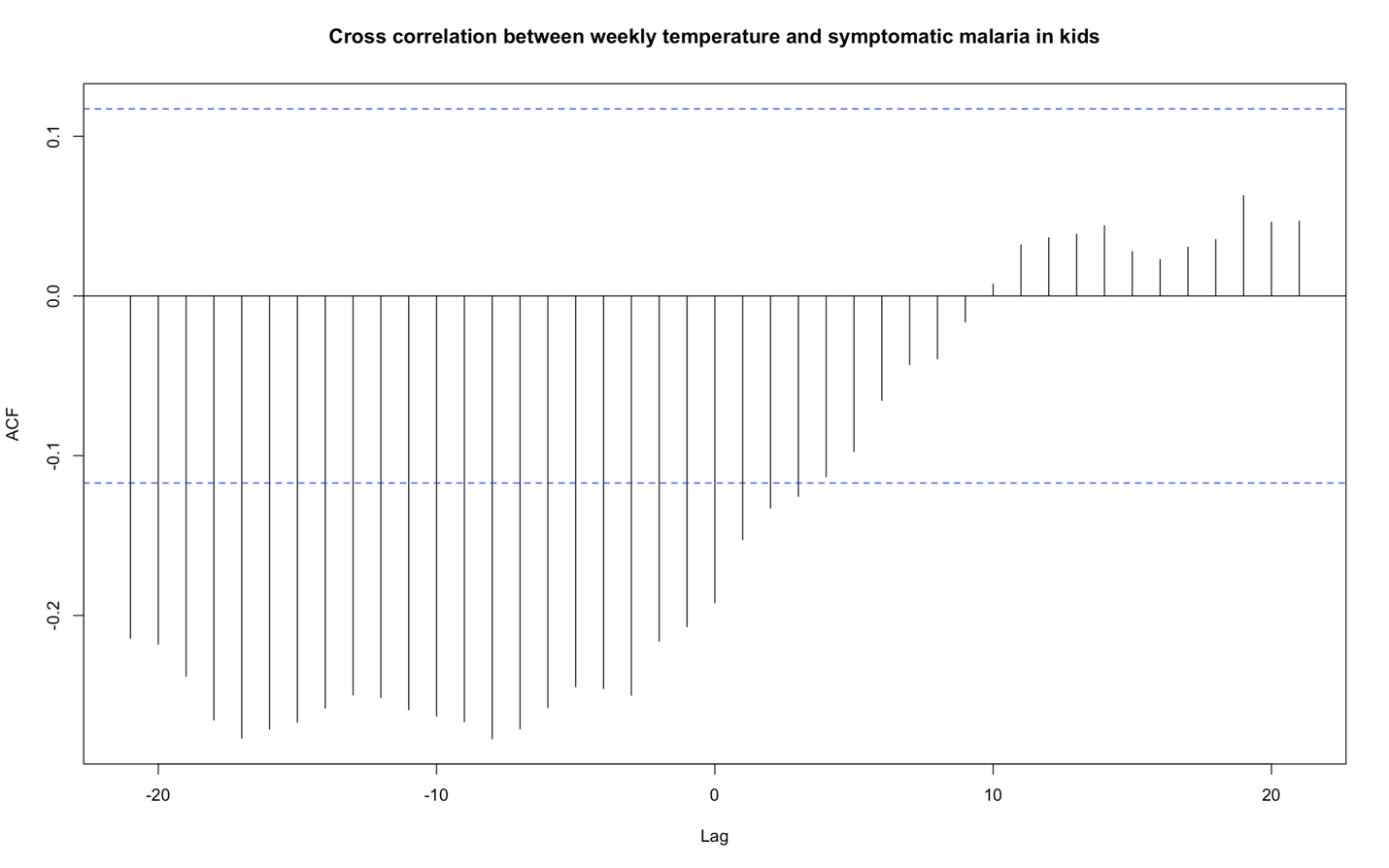


Fig 6: Cross correlation function between weekly average temperature and malaria cases

2014-2019 in Ukunda. Malaria cases occur after a lag of 0-21 weeks in temperature changes. A negative correlation is observed between temperature and malaria cases, indicating increasing temperature related to the decreasing in malaria case incidence.


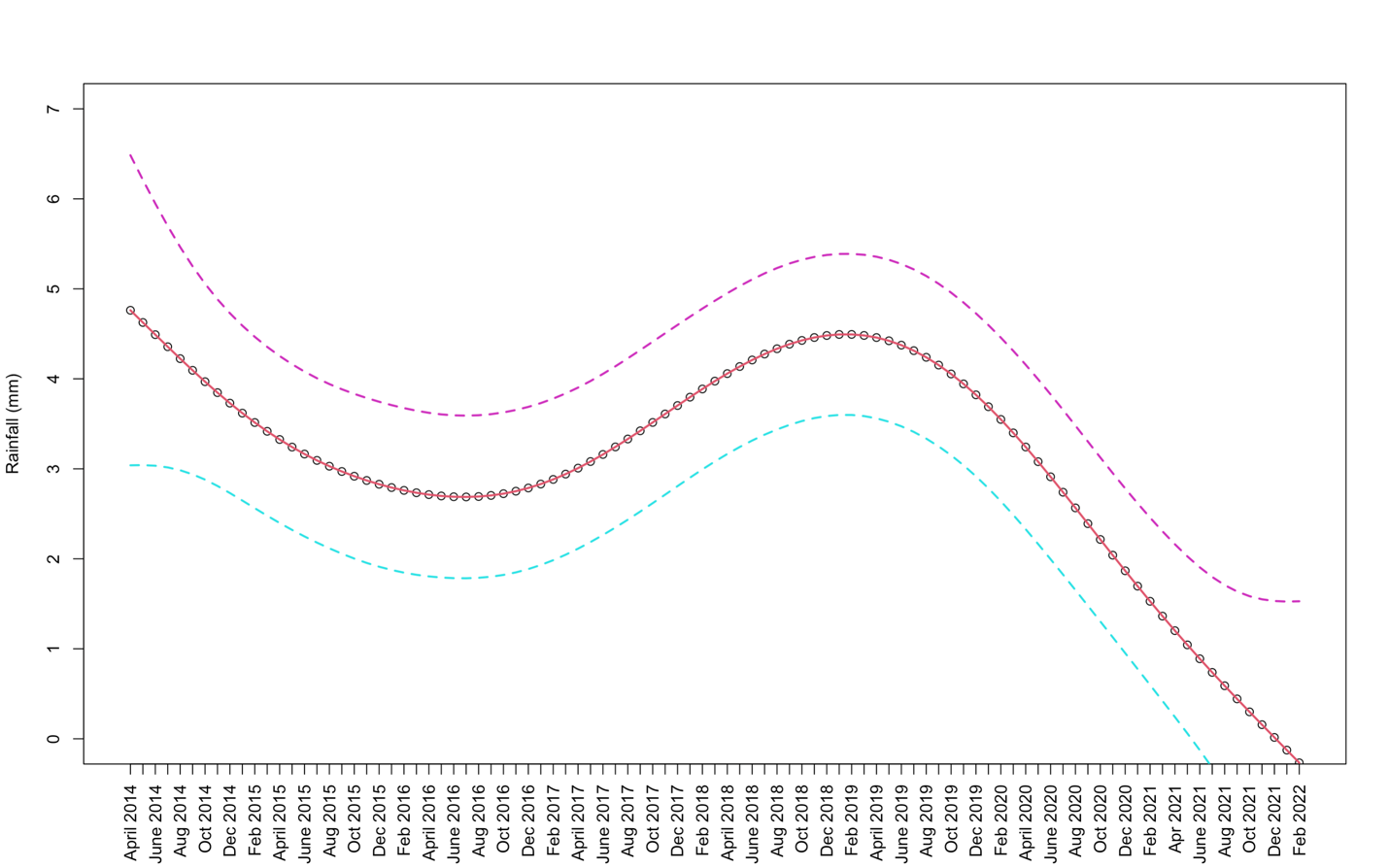


Fig 7: Generalized Additive Model fit to the rainfall data by the month in Ukunda 2014-2022. The red line shows the mean fit of the model and the magenta and blue dashed lines show the 95% CI for the mean model fit.


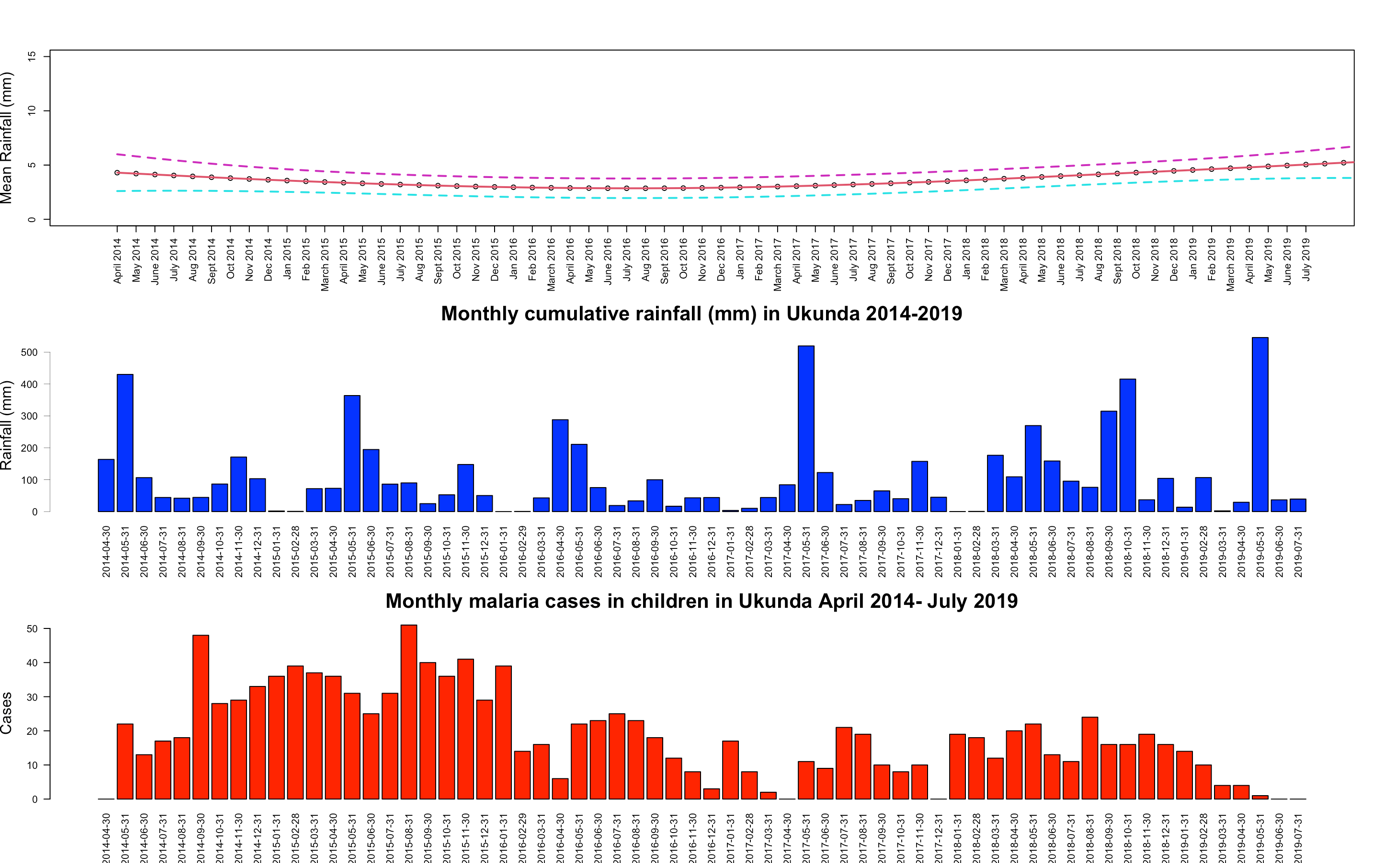


Fig 8: Panel a: Generalized Additive model (GAM) fitted to the rainfall data in Ukunda until 2019.

Panel b: Monthly sum of rainfall in Ukunda until 2019.

Panel c: Monthly malaria cases in Ukunda until 2019.


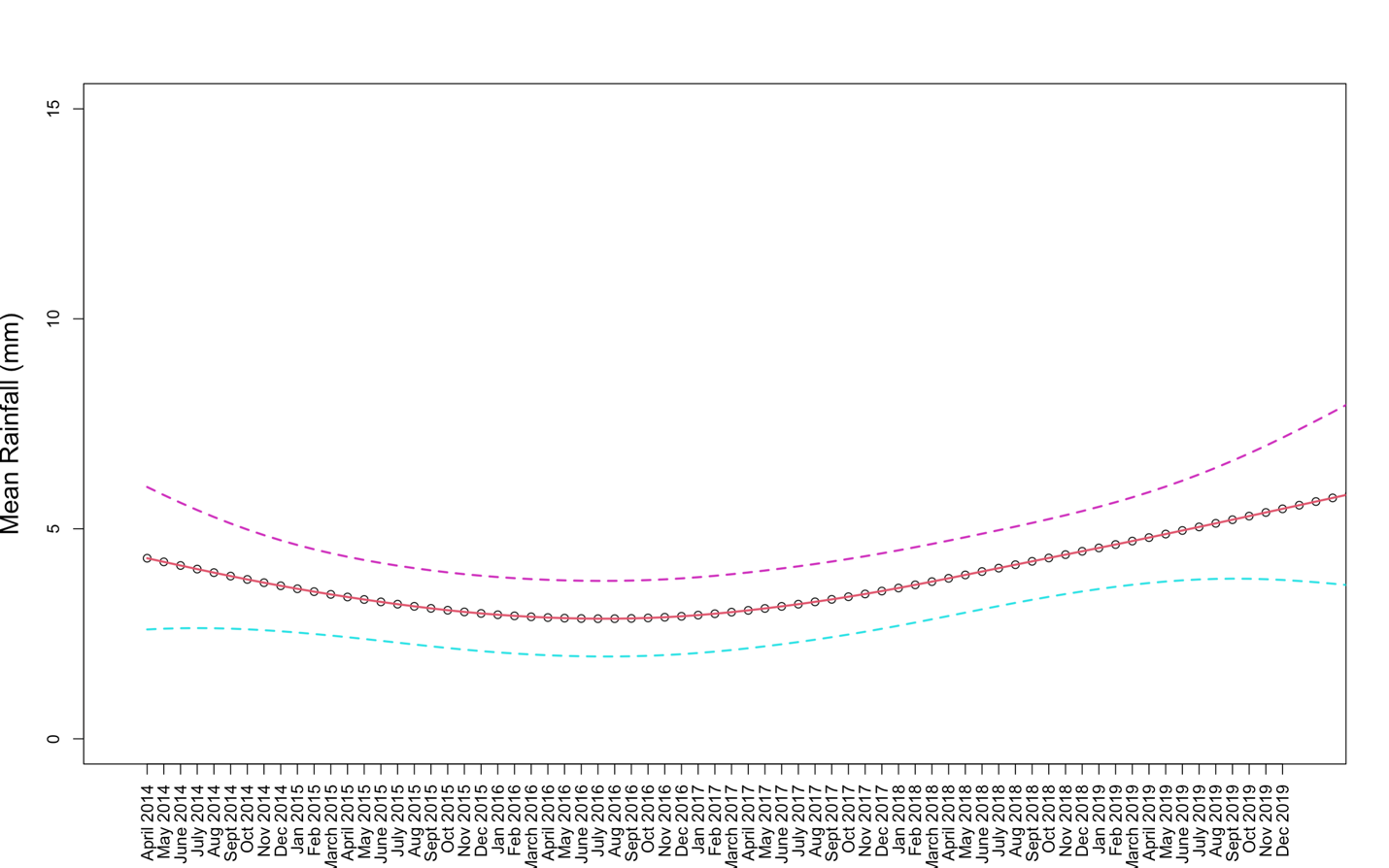


Fig 9: Generalized Additive Model fitted to the rainfall data by month until 2019 for Ukunda. The red line shows the mean fit of the model and the magenta and blue dashed lines show the 95% CI for the mean model fit.


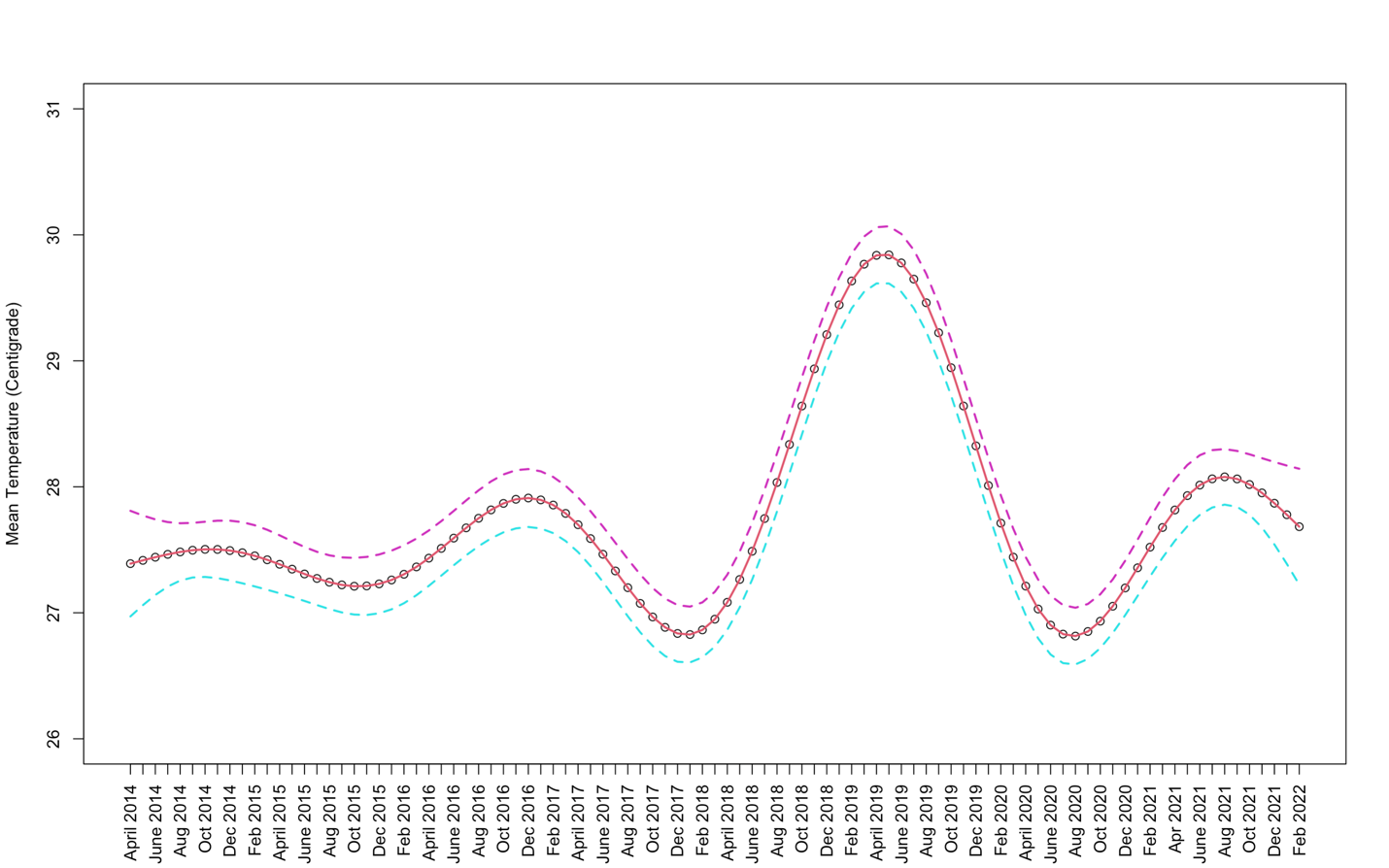


Fig 10: Generalized Additive Model fit to the rainfall data by the month in Ukunda 2014-2022. The red line shows the mean fit of the model and the magenta and blue dashed lines show the 95% CI for the mean model fit.
